# Extraction and Natural Bioactive Molecules Characterization in Spinach, Kale and Purslane: A Comparative Study

**DOI:** 10.3390/molecules26092515

**Published:** 2021-04-26

**Authors:** Boris Nemzer, Fadwa Al-Taher, Nebiyu Abshiru

**Affiliations:** 1 VDF FutureCeuticals, Inc., Momence, IL 60954, USA; bnemzer@futureceuticals.com (B.N.); Nebiyu.abshiru@futureceuticals.com (N.A.); 2 Department of Food Science and Human Nutrition, University of Illinois at Urbana-Champaign, Urbana, IL 61801, USA

**Keywords:** omega-3 fatty acids, spinach, kale purslane, phytochemicals, carotenoids, vitamins, minerals

## Abstract

Leafy green vegetables contain essential nutrients and are frequently consumed for their perceived health benefits. The purpose of this study was to profile the nutritional and natural bioactive phytochemical compounds extracted from freeze-dried spinach and kale and compare them with our previously published freeze-dried purslane results. Novel research suggests that these leafy greens contain an abundance of fatty acids, amino acids, organic acids, vitamins, and minerals, which can reduce the risk of many chronic diseases. LC-MS/MS screening identified 69 and 103 compounds in spinach and kale, respectively, including flavonoids, glucosinolates, and phenolic and organic acids. Out of a total of 26 flavonoids identified in the current study, only three were found in spinach. All three leafy greens showed nutritional and health benefits and the unique phytochemical compounds found only in purslane provide a strong basis to incorporate its distinct dietary benefits.

## 1. Introduction

Leafy green vegetables are important for a healthy diet as they promote the overall wellness of the human body. In addition to fiber, protein, and other macronutrients, they are a source of essential amino acids, organic acids, vitamins, minerals, and phytonutrients that have been reported to play essential roles in supporting human health [1]. Various nutritional elements, including vitamins and phytochemicals, found in vegetables also have antioxidant activity, which can protect against free radicals that damage cells in the human body. Diets rich in leafy greens that provide antioxidants and other phytonutrients, such as lutein and zeaxanthin, may play a role in mitigating oxidative damage in the body and age-related disorders, such as prevention of cancers, heart disease, Alzheimer’s, and eye disease [1].

Spinach and kale are among the most prevalent leafy greens in the United States and around the world, and published statistics demonstrate that they are grown and consumed in large quantities [2]. In 2018, it was reported that world production of spinach reached a staggering 26 million tons [3]. Kale has recently grown in popularity due to its marketing as a nutrient-dense “superfood” with functional food benefits [4,5]. In the United States, in 2012, over 6000 acres of kale were harvested, an increase of over 100% over just a five-year period from 2007 to 2012 [5]. The trends resulting in the increased growth and consumption of kale in the United States have continued to the present and are expected to continue in the future [4].

Statistics also support the conclusion that consumers are increasingly choosing to purchase and consume fresh greens compared with processed greens. This trend may result from consumer awareness and desire to obtain the perceived health benefits from the micronutrients and phytonutrients present in leafy greens when they are fresh or minimally processed, but which nutrients can easily degrade when heavily processed [6]. In 2000, the volume of vegetables consumed that were processed exceeded those that were fresh by more than 5% [6]. By 2017, the trend had reversed, with fresh vegetables now exceeding the volume of processed by almost 1% (comparing 2000, fresh: 47.3% and processed: 52.7% with 2017, fresh: 50.4% and processed: 49.6%) [6]. The change in consumer preferences towards minimally processed vegetables suggests that gentle processing methods that better preserve micronutrients—like freeze-drying—are on-trend and most appropriate for further study and chemical analysis.

Nowadays, consumers demand high-quality food products. Freeze-drying (FD) is performed by removing water from frozen food products under low pressure. FD extends the shelf-life of food products or food ingredients while maintaining quality. FD is known as the best technology to maintain the original phytonutrient composition in fruits and vegetables [7].

When assessed in parallel, it is apparent that spinach and kale contain an abundance of nutrients, phytochemicals and antioxidants [1]. The nutrients previously found in spinach and kale include vitamins A (beta-carotene), C, total ascorbic acid, K and B9 (folate) [8]. Spinach and kale are abundant with similar minerals (potassium, calcium, magnesium, sodium, phosphorus, and iron), with each possessing unique mineral profiles [8]. These nutrients and minerals are essential for normal biological functions. The most important phytochemicals in spinach and kale are carotenoids: β-carotene, lutein and zeaxanthin [8,9]. These antioxidant compounds are important in preventing chronic health diseases, such as cancer and heart disease [9]. The main phytochemicals found in kale with health benefits are glucosinolates, polyphenols, and carotenoids. Consumption of kale has been reported to relieve symptoms of gastric ulcers, treat diabetes mellitus, rheumatism, bone weakness, ophthalmologic problems, hepatic disease, anemia, and obesity. Additionally, similar to other cruciferous vegetables, kale showed antioxidant and anticarcinogenic potential [4].

Due to increasing consumer trends towards eating leafy greens, especially FD spinach and FD kale, and their concomitant health benefits, there is a need to quantify and compare their nutrients and phytochemicals. The aim of this paper is to profile the nutritional and natural bioactive phytochemical compounds extracted from freeze-dried spinach and kale and compare this with previously published data for freeze-dried purslane, an infrequently consumed and relatively unknown leafy green [10]. These data should further promote purslane as among the most nutrient-dense leafy greens and should recommend it for greater use as a health-promoting food ingredient in nutraceutical applications.

## 2. Results and Discussion

### 2.1. Fatty Acids Content

Spinach and purslane contained palmitic (C16:0), elaidic (C18:1cis), linoleic (C18:2n-6), and linolenic (C18:3n-3) fatty acids. C18:1cis was not identified in kale leaves; however, C16:0, C18:2n-6, and C18:3n-3 fatty acids were all identified. Comparison of the respective fatty acids in spinach and kale was conducted on a dry weight basis and revealed significant differences (<0.05) in C16:0 content of 2.99 ± 0.25 mg/100 g and 4.40 ± 0.18 mg/100 g. Additionally, when comparing omega-3 (C18:3n-3), spinach (10.84 ± 0.86 mg/100 g) and kale (16.69 ± 2.44 mg/100 g) contained significantly different (*p* < 0.05) amounts. However, no significant differences were observed for omega-6 (C18:2n-6) fatty acids between spinach (3.48 ± 0.43 mg/100 g) and kale (3.54 ± 0.61mg/100 g).

Fatty acids profile of spinach and kale compared to published data for purslane are shown in Table 1.

It can be seen that purslane is enriched with a whole profile of fatty acids and has 10× more total fatty acids than either spinach or kale. Purslane was found by Nemzer et al. [10] to contain the highest abundance of omega-6 and omega-3 fatty acids (37.78 ± 1.97 mg/100 g and 98.35 ± 6.78 mg/100 g, respectively) when compared to omega-3 in spinach and kale (10.84 ± 0.86 mg/100 g and 16.69 ± 2.44 mg/100 g, respectively) and omega-6 in spinach (3.48 ± 0.43 mg/100 g) and kale (3.54 ± 0.61mg/100 g).

By analyzing the total fatty acid content of each material on a dry weight basis, this investigation showed the total spinach leaves’ amount to be 18.67 mg/100 g and had 16.01% and 83.88% of saturated and unsaturated fatty acid content, respectively, of the total fatty acid content. The total kale leaves’ fatty acid amount was found to be 23.61 mg/100 g, dry weight, and had 18.64% and 81.36% of saturated and unsaturated fatty acid content, respectively, of the total fatty acid content. In our previous study [10], the total fatty acids content in purslane was a whole order of magnitude higher at 191.83 mg/100 g, dry weight, displaying ratios of 21.12% and 78.88% of saturated and unsaturated fatty acid content, respectively, of the total fatty acid content.

This study found the polyunsaturated fatty acids (PUFA)/saturated fatty acids (SFA) ratio for spinach and kale leaves to be 4.79 and 4.59, respectively. Nemzer et al. [10] observed the PUFA/SFA ratio in the purslane plant as 3.36.

Ayaz et al. [11] noticed that the most abundant fatty acid in kale was C18:3n-3 comprising 54.0%, followed by C18:2n-6 and C18:0, each encompassing 11.8% of total fatty acids. In comparison, our study showed the highest abundance of C18:3n-3 in kale comprising 82.1% of total fatty acids, followed by C16:0 at 17.9% and C18:2-6 at 14.4% of the total fatty acids. Simopoulos [12] noticed that omega-3 in spinach was 52.4% of the total fatty acid content of 170 mg/100 g, wet weight. Our results show that C18:3n-3 was 57% of the total fatty acids in spinach of 18.7 mg/100 g, dry weight. The differences are attributed to the different water content. Nemzer et al. [10] showed purslane contains 51.3% of C18:3n-3, 19.7% of C18:2n-6, and 17.8% of C16:0 of the total fatty acids of 191.83 mg/100 g, dry weight.

Omega-6 (C18:2n-6) and omega-3 (C18:3n-3) fatty acids are vital to the human diet because they can’t be synthesized by humans. A balance of omega-6/omega-3 fatty acids is advantageous for treating and preventing coronary artery disease, hypertension, diabetes, arthritis, osteoporosis, other inflammatory and autoimmune disorders, and cancer. Consequently, without the adequate intake from the diet, nutritional deficiency can result [12]. The omega-6/omega-3 ratios were found to be low (1:3 and 1:5) for spinach and kale, respectively. These indicate higher amounts of omega-3, which is desirable to prevent inflammation and chronic diseases. Similarly conforming to this recommendation, a ratio of omega-6/omega-3 (1:3) was shown in the cultivated purslane in our previous study [10]. Low ratios of omega-6 to omega-3 are desirable in reducing the risk of many chronic diseases [12]. This demonstrates that regular inclusion of purslane as well as spinach and kale in the diet has the potential to confer benefits for health outcomes.

### 2.2. Phytochemicals Identification

**Figure 1:**
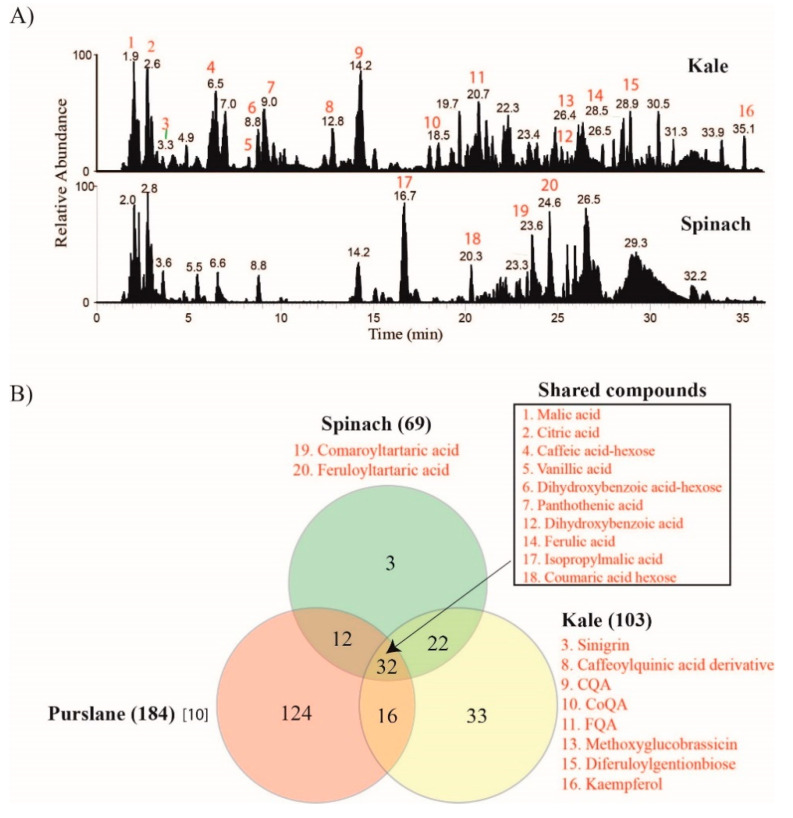
Comparative phytochemicals content analysis of leafy greens by LC-MS/MS. (**A**) Comparison of total ion chromatograms of kale (top panel) and spinach (bottom panel). (**B**) Venn diagram showing the number of compounds identified in kale (total 103; unique 33), spinach (total 69; unique 3) and purslane (total 184; unique 124) and those shared among the greens.

This analysis aims at profiling the phytochemical contents of kale and spinach, as well as comparing this result with published data for purslane. High-resolution tandem mass spectrometry analyses were performed on extracts of spinach and kale to identify specific compounds and groups of compounds. A total of 118 compounds representing organic acids, phenolic acids, flavonoids, glucosinolates, chlorogenic acid, amino acids, and vitamins were identified in both spinach and kale in Table S1 (Supplementary Material). The LC/MS base peak chromatogram (BPC) representing each extract is shown in Figure 1A. The BPC profile shows that fewer number of peaks were detected in spinach compared to kale. The number of identifications in spinach and kale extracts with manually confirmed fragment spectra are 69 and 103, respectively (Figure 1B). In contrast, 184 phytochemicals were previously reported in purslane [10].

**Figure 2:**
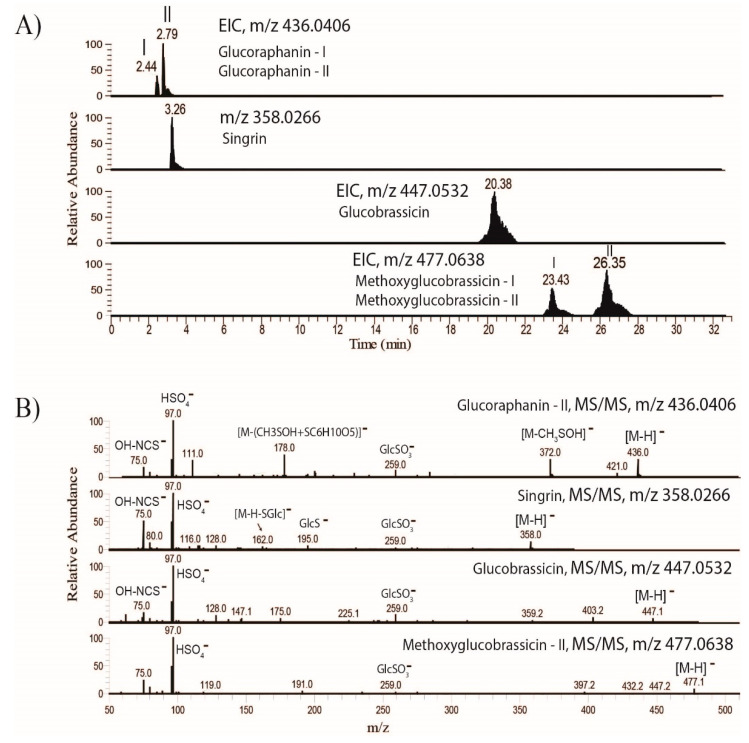
Representative LC-MS/MS profiles of kale glucosinolates. (**A**) Extracted ion chromatograms (EIC) and (**B**) the corresponding fragmentation spectra of glucoraphanin sinigrin, glucobrassicin, and methoxyglucobrassicin.

Comparison of the number of identifications in spinach and kale with those of previously published data for purslane [10] revealed the number of unique phytochemicals as follows: 124 for purslane, 33 for kale, and three for spinach (Figure 1B). Among the major purslane phytochemicals that were missing in spinach and kale, 25 are alkaloids and more than 56 cinnamoyl-organic acid conjugates (Table S1). Spinach, kale, and purslane shared 32 compounds, including malic, citric, vanillic, pantothenic, dihydroxybenzoic and ferulic acids [10]. The number of compounds shared between kale and spinach, kale and purslane [10], and spinach and purslane [10] are, respectively, 22, 16, and 12. This study identified a total of 11 glucosinolates in kale (Table S1). These compounds were absent in spinach and were not reported in purslane extract screened by Nemzer et al. [10]. A number of studies have previously reported high levels of glucosinolates in kale [13]. Similarly, the current study identified several glucosinolates, including, in increasing order of relative peak intensity, sinigrin, methoxyglucobrassicin, glucobrassicin, 4-hydroxyglucobrassicin, glucoraphanin, progoitrin, gluconasturtin, and neoglucobrassicin. Representative extracted ion chromatograms (EIC) and fragment spectra of four kale glucosinolates are shown in Figure 2.

Glucoraphanin and Methoxyglucobrassicin were detected in two different isoforms (I and II), as shown in Figure 2A. Dissociation of the glucosinolates generated common fragment ions at *m*/*z* 75.0, 97.0, and 259.0, corresponding to, respectively, OH-NCS-, HSO_4_^−^ and GlcSO_3_^−^.

Moreover, the current study identified 3 and 26 flavonoid compounds in spinach and kale, respectively (Table S1). In comparison, Nemzer et al. [10] reported 30 flavonoid compounds in purslane. Interestingly, some of the flavonoids identified in kale have structural features that are different from those reported in purslane. The flavonoids identified in purslane are mainly non-acylated with one or two glycoside attachments. However, almost half of the flavonoids identified in kale are structurally more complex, having large sugar moieties and acylated with ferulic, caffeic, coumaric, or sinapinic acid. Such complex flavonoids in kale were previously reported [14], and it has been suggested that the varieties of glycosylation and acylation patterns might potentially modulate the health-promoting properties of the flavonoid glycosides.

### 2.3. Total Carotenoids Content

Statistically significant differences (*p* < 0.05) in total carotenoids were observed between the spinach (0.346 ± 0.042 mg/g, dry weight) and kale leaves (0.454 ± 0.045 mg/g, dry weight). Nemzer et al. [10] found that when compared to either, however, purslane had by far the highest total carotenoids content (1.21 ± 0.06 mg/g, dry weight) based on lutein. Carotenoids have antioxidant and photoprotective properties that may be useful for preventing or treating age-related macular degeneration [9].

Spinach grown in the U.S. showed a lutein content range of a high of 0.1298 mg/g and a low of 0.0649 mg/g of fresh mass when a total of 13 commercial spinach cultigen seeds were grown under similar conditions [9].

### 2.4. Minerals Content

Statistically significant differences (*p* < 0.05) existed for all pairwise multiple comparisons for each mineral between spinach and kale (Table 2).

Similar types of minerals with high abundances found in the leafy greens were potassium (K), magnesium (Mg), calcium (Ca), and phosphorus (P). The most abundant minerals found in this study for spinach in order of highest concentration were K, Ca, Mg and P, and, for kale, K, Ca, P and Mg.

Kale contained more Ca and P (1700.00 ± 20.0 mg/100 g, dry weight, and 549.00 ± 3.00 mg/100 g, dry weight, respectively) than spinach (803.33 ± 10.12 mg/100 g, dry weight, and 290.67 ± 1.15 mg/100 g, dry weight, respectively). In a study performed by Ayaz et al. [11], the highest macronutrient mineral in kale (*Brassica oleraceae* L. var. *acephela* DC) was determined to be Ca (19.7 ± 0.6 mg/g, dry weight), K (13.5 ± 0.7 mg/g, dry weight) and P (5.73 ± 0.9 mg/g, dry weight) and the highest micronutrient mineral was Fe (72.6 µg/g, dry weight), Mn (53.5 ± 1.9 µg/g, dry weight), and Zn (39.4 ± 1.2 µg/g, dry weight).

Table 2 lists the mineral amounts detected in spinach and kale in this study. Purslane results are recorded from our previous study [10] for comparison. Purslane had greater amounts of Ca (914.33 ± 17.95) than that for spinach (803.33 ± 10.12) and less than for kale (1700.00 ± 20.0). Spinach had more Na (866.33 ± 6.51 mg/100 g, dry weight) compared to kale (45.80 ± 0.92 mg/100 g, dry weight) or purslane (35.07 ± 1.72 mg/100 g, dry weight) [10]. Purslane comprised higher levels of Mg and K (1266.67 ± 20.82 and 6400.00 ± 141.07 mg/100 g, dry weight, respectively) [10] than spinach (286.33 ± 8.96 and 1503.33 ± 46.19 mg/100 g, dry weight, respectively) and kale (362.67 ± 2.08 and 4223.33 ± 75.06 mg/100 g, dry weight, respectively). Purslane has been shown to have a muscle relaxant effect because of the high potassium concentration [15].

### 2.5. Amino Acids Content

Kale contained the highest amount of glutamic acid, aspartic acid, and leucine (3747 ± 45.09, 3017 ± 47.26, and 2593 ± 23.09 mg/100g, dry weight, respectively) compared to spinach (2630 ± 20.00, 1987 ± 20.82, and 1770 ± 17.32 mg/100 g, dry weight, respectively). Essential amino acids such as lysine, threonine, phenylalanine, isoleucine, valine, and methionine were detected in considerable amounts in kale and lesser amounts in spinach. Other non-essential amino acids (serine, proline, glycine, tyrosine, arginine, and cysteine) were also found in the three leafy greens.

Table 3 displays amino acid content in spinach and kale determined in this study, and previously published purslane results [10] are also listed for comparison.

In general, the most abundant amino acids detected in all three leafy greens with statistically significant differences (*p* < 0.05) were glutamic acid, aspartic acid, and leucine. Overall, purslane contained the lowest amount of these amino acids when compared to spinach and kale, as can be seen from Table 3.

Ayaz et al. [11] also noted that the most abundant amino acid found in kale leaves was glutamic acid, which was detected at 33.2 ± 1.1 mg/g, dry weight, and contributing 12.2% of the total amino acid content, followed by aspartic acid at 27.6 ± 0.8 mg/g, dry weight, which made up 10.2% of the total amino acid content and then arginine at 20.6 ± 0.8 mg/g, dry weight, and leucine at 20.3 ± 0.6 mg/g, dry weight, at 7.6% and 7.5% of the total amino acids, respectively. In a study performed by Lisiewska et al. [16], the major amino acids in kale leaves with midribs removed were glutamic acid, proline, and aspartic acid, and the proportion of the total amino acid content was 12%, 12%, and 10%, respectively. In the present study, the ratios for the greatest abundances of amino acids were 14%, 9.4%, and 11% for glutamic acid, aspartic acid, and leucine, respectively, in kale. Ayaz et al. [11] observed the presence of other amino acids in lower quantities in kale, which ranged between 3 and 9 mg/g, dry weight, in cysteine, histidine, methionine, and tryptophan and accounted for about 1–3% of the total amino acids. In this study, these other amino acids ranged between 3 and 7 mg/g, dry weight, in kale. Tryptophan, however, was not tested.

The current investigation found the proportion of glutamic acid and aspartic acid to total amino acids in spinach to be 13.9% and 10.5%, dry weight, respectively. Lisiewska et al. [17] determined the major amino acids in spinach to be glutamic acid and aspartic acid (12% and 11% of the total amino acids content, respectively).

The proportion of essential amino acids in total amino acids for this investigation was 41% and 42%, respectively, for spinach and kale. For purslane, Nemzer et al. [10] noted the ratio to be 41%. Lisiewska et al. [16] reported this ratio to be 44% and 43%, respectively, for fresh and cooked kale leaves and 49% for spinach [17], which is consistent with this study.

### 2.6. Vitamins Content

Vitamin A, as retinol, was not detected in spinach or kale. Statistically significant different (*p* < 0.05) amounts of Vitamin C (ascorbic acid) were detected in spinach (51.9 ± 1.5 mg/100 g, dry weight) and kale (135.0 ± 2.6 mg/ 100 g, dry weight). No statistically significant difference (*p* < 0.05) existed for the antioxidant Vitamin E content between spinach and kale in this study.

Table 4 shows the quantities of vitamins determined in this study. Results for purslane from a previous study [10] are also listed for comparison.

Similar to spinach and kale, no Vitamin A was detected in purslane [10], but the highest abundance of Vitamin C (152 ± 9.3 mg/100 g, dry weight) was detected compared to the content in spinach and kale. Additionally, purslane [10] contained significantly higher amounts of almost three orders of magnitude of Vitamin E (11.967 ± 57.7 µg/100 g, dry weight) than either spinach (13.5 ± 0.7 µg/100 g, dry weight) or kale (14.2 ± 0.2 µg/100 g, dry weight) in this study. Vitamin E may have a therapeutic and/or preventative role in brain aging, cognition, and Alzheimer’s disease [18].

Sikora and Bodziarczyk [19] determined a Vitamin C average content of 62.27 ± 13.72 mg/100 g for three years of cultivar of raw kale leaves. The results of the current study showed the Vitamin C content for kale to be higher (135.0 ± 2.6 mg/100 g, dry weight). From the 27 spinach cultivars investigated [20], the average value reported for Vitamin C was 48.61 ± 6.05 mg/100 g, fresh weight, for organic plants and 35.43 ± 6.08 mg/100 g, fresh weight, for conventional crops. Results from this study showed 51.9 ± 1.5 mg/100 g, dry weight, of ascorbic acid in spinach, which was in the range of what was reported previously. Genotypes and farming practices can influence the levels of ascorbic acid in spinach and may account for the ranges noted here. By comparison, Nemzer et al. [10] showed the richest source of ascorbic acid is purslane (152 ± 9.3 mg/100 g, dry weight), which was much higher than spinach.

### 2.7. Organic Acids Content

Table 5 shows organic acid results for spinach and kale and previous results for purslane [10].

The amount of citric acid and malic acid in kale (4826.44 ± 22.55 and 6138.00 ± 19.25 mg/100 g, dry weight, respectively), were greater than that for spinach (1031.94 ± 29.42 mg/100 g, dry weight for citric acid and not detected for malic acid). Previous data determined purslane to contain citric acid (510.08 ± 2.17 mg/100 g, dry weight) and malic acid (1155.38 ± 10.72 mg/100 g, dry weight). These values are higher than the results reported by Ayaz et al. [11] for kale leaf, where the contents of major organic acids for citric acid and malic acid were found to be 2213 and 151 mg/100 g, dry weight, respectively.

Gonnella et al. [21] noted that anti-nutritive compounds, such as oxalates, could be formed in high amounts in plants. These inhibit nutrient absorption, especially minerals such as calcium and iron, that can cause kidney stones and gout. Nemzer et al. [10] noticed that the most abundant organic acids detected in the whole purslane were oxalic acid, followed by malic acid and citric acid. However, this study determined that the oxalate content in spinach (8387.47 ± 14.87 mg/100 g, dry weight) was higher than that in purslane [10] (6757.63 ± 12.83 mg/100 g, dry weight). No oxalic acid was observed in kale.

## 3. Materials and Methods

### 3.1. Raw Materials and Chemicals

Kale was grown from seed at Van Drunen Farms, VDF (Momence, IL, USA). Planted in April 2019, the kale was conventionally grown using Good Agricultural Practices and machine harvested at approximately 50 days. Harvested kale was immediately washed and frozen prior to freeze-drying by VDF.

Baby spinach was purchased from a local grocery store in Momence, IL, in December 2019 and was freeze-dried.

All samples were ground to a fine powder using IKA A11 basic analytical mill. Freeze-dried kale and spinach samples were tested for similar chemical composition. All chemicals and reagents used in this study were of analytical grade.

### 3.2. Fatty Acids Analysis

About 100 mg of freeze-dried spinach and kale were extracted for fatty acids by an acid-catalyzed reaction (AOAC Official Method 996.06 (modified)) [22,23]. An internal standard, C13:1(methyl-12-tridecenoate; Nu-Chek Prep, Inc., Elysian, MN, USA), was added to the samples. Samples were saponified with 0.02 N NaOH in methanol by heating (70 °C for 1 h), and then the fatty acids were converted to fatty acid methyl esters (FAMEs) with 13–15% boron trifluoride in methanol by heating (70 °C for 1 h). The FAMEs were extracted by adding 5 mL of 0.01 M NaCl in water to each of the samples, followed by 5 mL of hexane. The layers separated, and the organic (top) layer containing the FAMEs was transferred into a vial for gas chromatography (GC) analysis.

The FAME content of freeze-dried spinach and kale extracts was separated on an HP-88 (60 m × 0.250 mm, 0.2 µm) fused silica column and determined by an Agilent 7890A Gas Chromatography equipped with a flame ionization detector. Identification and quantitation were performed by comparing the relative retention times of FAME peaks of the analyzed samples with those of commercial standards (FAMEs reference standard mixture 37, Sigma-Aldrich, St. Louis, MO, USA). The concentration of each FAME was calculated by plotting the ratio of the peak area of the FAME in the standards to the peak area of the internal standard against the ratio of the concentration of the FAME to the concentration of the internal standard. FAMEs were converted to fatty acids as outlined in the AOAC Official Method 996.06 (modified). The composition of the fatty acids was expressed as mg/100g for each fatty acid detected.

### 3.3. Phytochemicals Analysis

Three grams of freeze-dried spinach and kale were sonicated in 50 mL of 80% methanol (60 °C for 1 h), centrifuged (9000 rpm for 5 min), and dried to 1 mL under N2 spray for extraction of phytochemicals. The extracts were then filtered through a 0.2 µm PTFE, and 100 µL of the filtrate was diluted to 1 mL with 50% methanol in water prior to injection.

LC-MS/MS data of the phytochemicals content of spinach and kale extracts were obtained from a Q-Exactive Hybrid Quadrupole-Orbitrap mass spectrometer (Thermo Scientific, Waltham, MA USA) coupled with a Dionex UltiMate 3000 UHPLC system (Thermo Scientific, Waltham, MA, USA). A C18 analytical column (Agilent Poroshell 120 EC-C18, 3 × 150 mm, 2.7 µm) was used to separate the compounds into a linear gradient from 0 to 70% ACN (containing 0.1% formic acid) at 0.4 mL/min for over 60 min. The MS instrument was operated in a positive or negative ion mode and a capillary voltage of 3.2 kV. Precursor ions were scanned in the range of 100–1200 *m*/*z* at a resolution of 70,000 and an automatic gain control target value of 1.0 × 106. Precursor ions were fragmented in the higher-energy collisional activated dissociation cell, and the fragments were analyzed in the orbitrap analyzer. Thermo Scientific Compound Discover 3.0 software program was used to search multiple databases for the identification of polyphenols.

### 3.4. Total Carotenoid Analysis

Approximately 0.4 g of freeze-dried spinach and kale plants were extracted with 10 mL of water-saturated butanol for total carotenoids using the AACCI method 14–50.01 (modified) [24]. The mixture was covered and allowed to settle for an hour, then shaken and allowed to settle again for another hour at room temperature. Thereafter, the samples were centrifuged (14,000 rpm and 20 °C for 10 min). The total carotenoids were measured with a UV spectrophotometer and were quantified against lutein (HPLC grade, >90% purity, PhytoLab GmbH & Co. KG, Vestenbergsgreuth, Germany). The total carotenoid content for each sample was calculated in mg/g.

### 3.5. Minerals Analysis

Freeze-dried spinach and kale plants were digested with concentrated nitric acid and water according to AOAC methods 984.27 and 985.01 (modified) [23]. After digestion, the samples were brought to a final volume with water. Calcium, copper, iron, magnesium, manganese, phosphorus, potassium, sodium, and zinc were identified and quantified by inductively coupled plasma-optical emission spectrometry (ICP–OES). The results for each mineral were calculated in mg/100 g.

Selenium in the samples was identified and quantified by ICP mass spectrometry (ICP–MS) per modified AOAC Official Method 2011.19, and the amount was determined by comparing the amounts generated by the unknown to those generated by standard solutions. The results for selenium were calculated in µg/100 g [23].

### 3.6. Total Amino Acids Analysis

Freeze-dried spinach and kale samples were hydrolyzed in 6 N hydrochloric acid at about 110 °C for 24 h. To prevent halogenation of tyrosine, phenol was added to the acid. Cystine was derivatized during hydrolysis in hydrochloric acid containing dithiodipropionic acid to S-2-carboxyethylthiocysteine. Amino acids were derivatized with two different reagents, o-phthalaldehyde for the primary amino acids and fluorenylmethyl chloroformate for the secondary amino acids, before injection and analysis on the HPLC [25,26].

### 3.7. Vitamins Analysis

Freeze-dried spinach and kale samples were extracted and analyzed for vitamins A, C, and E as follows:

Vitamin A (Retinol): Samples underwent alkali hydrolysis to break down fat and release the vitamins. Vitamin A was extracted with an organic solvent and quantified as retinol by HPLC per modified AOAC methods 992.04, 992.06, and 2001.13 [23].

Vitamin C (Ascorbic acid): Vitamin C was extracted from the samples with an m-phosphoric acid and acetic acid solution. It was separated on an HPLC system and quantitated with a UV detector per AOAC Official Method 967.22 (modified) [23].

Vitamin E (alpha Tocopherol): Vitamin E was extracted from the samples by alkali hydrolysis [27,28,29]. Tocopherols were extracted with an organic solvent, run on a HPLC system, and the amount was determined using fluorescence detection.

### 3.8. Organic Acid Analysis

Organic acids were extracted from freeze-dried spinach and kale samples with 0.2 M potassium phosphate buffer (pH 2.4). They were subsequently separated on an HPLC system and analyzed with a UV detector per AOAC method 986.13 [23].

### 3.9. Statistical Analysis

At least four replicates were extracted and tested for each analysis. Microsoft Excel 365 ver. 1908 was used to calculate the mean and standard deviation (SD). Data were reported on a dry weight basis as the mean ± SD. Comparisons of results between the samples were determined using analysis of variance (ANOVA) with Sigma Plot 14.0 (Systat Software, San Jose, CA, USA) to determine significant differences at a 5% level of significance.

## 4. Conclusions

While spinach has been known for many years to contain health benefits, kale has gained popularity in recent years as a “superfood.” This study examined the scientific evidence to compare the nutritional value of these leafy green vegetables to our previously published study on purslane [10].

This investigation showed that freeze-dried purslane contained significantly higher amounts of omega-3 and is enriched with the highest profile of total fatty acids (saturated and unsaturated) than either freeze-dried spinach or kale. Purslane includes omega-3, omega-6, and omega-9 (3:1:0.5), which all provide health benefits. Similar to purslane [10], a lower ratio of omega-6/omega-3 (1:3) in spinach was also identified and is recommended. Kale had a 1:5 ratio.

Kale had the greatest amount of calcium and phosphorus compared to purslane and spinach, whereas purslane contained significantly more magnesium and potassium than either kale or spinach. While all three leafy greens were rich in glutamic acid, aspartic acid, and leucine, the concentrations were different. The greatest number of these amino acids was detected in kale, followed by spinach and purslane. Purslane contained the highest amount of antioxidants, Vitamin C and E, compared to spinach and kale. Kale had significantly more malic acid than purslane, while spinach did not have malic acid. The content of citric acid was greatest in kale, followed by spinach and then purslane [10].

This study reported that spinach contains significantly higher levels of the anti-nutritive, oxalic acid, than purslane [10], while in kale, none was detected. The leafy green vegetables (spinach, kale, and purslane) can be used in combination or as single food ingredients, especially for functional and nutraceutical applications. These analyses support the conclusion that diets abundant in these leafy greens have the potential to delay the onset of age-related diseases.

Purslane is uniquely enriched with derivatives of phenolic-organic acid conjugates, alkaloids, and non-acylated flavonoids, as well as select vitamins and nutritional minerals. Kale is differentiated by containing high levels of glucosinolates and acylated flavonoids. Additionally, it is strongly suggested that complementary nutritional benefits of spinach, kale, and purslane could be achieved through the selective formulation of these freeze-dried powders.

## Figures and Tables

**Table 1:** Fatty acid content in spinach, kale, and purslane [10]. (Mean mg/100 g ± SD; n = 4), dry weight.

Fatty Acids	Spinach	Kale	Purslane [10]
C16:0 (saturated, palmitic acid)	2.99 ± 0.25 ^a^	4.40 ± 0.18 ^b^	34.05 ± 1.91 ^c^
C16:1 (palmitoleic acid, Omega-7)	ND	ND	ND
C18:0 (saturated, stearic acid)	ND	ND	6.46 ± 0.48
C18:1 *cis* (elaidic acid, Omega-9)	1.34 ± 0.18 ^a^	ND	15.19 ± 1.55 ^b^
C18:2 *cis* (linoleic acid, Omega-6)	3.48 ± 0.43 ^a^	3.54 ± 0.61 ^b^	37.78 ± 1.97 ^c^
C18:3n-3 *cis* (linolenic acid, Omega-3)	10.84 ± 0.86 ^a^	16.69 ± 2.44 ^b^	98.35 ± 6.78 ^c^
Total SFA	2.99	4.4	40.51
Total MUFA	1.34	ND	15.19
Total PUFA	14.32	19.21	136.13
Total Fatty Acids	18.67	23.61	191.83

ND-Not detected. Note: Values followed by different letters denote significant difference at *p* < 0.05 for each analyte between spinach, kale, and purslane across rows; those followed by same letters denote no significant difference. SFA: saturated fatty acids; MUFA: monounsaturated fatty acids; PUFA: polyunsaturated fatty acids.

**Table 2:** Mineral content in spinach, kale, and purslane [10] (Mean ± SD; n = 3), dry weight.

	Spinach	Kale	Purslane
Calcium (mg/100 g)	803.33 ± 10.12 ^b^	1700.00 ± 20.0 ^c^	914.33 ± 17.95 ^a^
Copper (mg/100 g)	0.82 ± 0.00 ^b^	0.60 ± 0.01 ^c^	1.18 ± 0.02 ^a^
Iron (mg/100 g)	7.16 ± 0.03 ^b^	9.75 ± 0.07 ^c^	41.73 ± 0.67 ^a^
Magnesium (mg/100 g)	286.33 ± 8.96 ^b^	362.67 ± 2.08 ^c^	1266.67 ± 20.82 ^a^
Manganese (mg/100 g)	2.19 ± 0.01 ^b^	5.74 ± 0.04 ^c^	6.77 ± 0.12 ^a^
Phosphorus (mg/100 g)	290.67 ± 1.15 ^b^	549.00 ± 3.00 ^c^	281.67 ± 3.21 ^a^
Potassium (mg/100 g)	1503.33 ± 46.19 ^b^	4223.33 ± 75.06 ^c^	6400 ± 141.07 ^a^
Sodium (mg/100 g)	866.33 ± 6.51 ^b^	45.80 ± 0.92 ^c^	35.07 ± 1.72 ^a^
Zinc (mg/100 g)	5.17 ± 0.04 ^b^	3.35 ± 0.01 ^c^	6.62 ± 0.18 ^a^
Selenium (µg/100 g)	<2.5 ± 0.00 ^b^	3.78 ± 0.05 ^c^	6.63 ± 0.20 ^a^

Note: Values followed by different letters denote significant difference at *p* < 0.05 for each analyte between spinach, kale, and purslane across rows; those followed by the same letters denote no significant difference.

**Table 3:** Amino acid content in spinach, kale, and purslane [10] (Mean mg/100 g ± SD; n = 3), dry weight.

Amino acids	Spinach	Kale	Purslane
Aspartic acid	1987 ± 20.82 ^b^	3017 ± 47.26 ^c^	1640 ± 0 ^a^
* Threonine	952 ± 10.02 ^b^	1417 ± 11.55 ^c^	759 ± 5.51 ^a^
Serine	858 ± 3.51 ^b^	1247 ± 11.55 ^c^	756 ± 5.29 ^a^
Glutamic acid	2630 ± 20.00 ^b^	3747 ± 45.09 ^c^	2187 ± 11.55 ^a^
Proline	943 ± 13.20 ^b^	1360 ± 26.46 ^c^	824 ± 2.08 ^a^
Glycine	1153 ± 5.77 ^b^	1573 ± 11.55 ^c^	918 ± 7.55 ^a^
Alanine	1187 ± 5.77 ^a^	1730 ± 20.00 ^b^	1213 ± 15.28 ^a^
* Valine	1153 ± 11.55 ^b^	1727 ± 15.28 ^c^	976 ± 9.85 ^a^
* Isoleucine	902 ± 12.58 ^b^	1320 ± 10.00 ^c^	801 ± 9.17 ^a^
* Leucine	1770 ± 17.32 ^b^	2593 ± 23.09 ^c^	1463 ± 5.77 ^a^
Tyrosine	905 ± 11.36 ^a^	1197 ± 11.55 ^b^	677 ± 1.73 ^a^
* Phenylalanine	1113 ± 5.77 ^b^	1663 ± 20.82 ^c^	853 ± 7.51 ^a^
* Lysine	928 ± 15.00 ^b^	1813 ± 25.17 ^c^	978 ± 13.50 ^a^
* Histidine	481 ± 3.21 ^b^	650 ± 1.73 ^c^	346 ± 3.21 ^a^
Arginine	1313 ± 101.6 ^b^	1770 ± 10.00 ^c^	957 ± 2.00 ^a^
Cystine	266 ± 8.08 ^b^	295 ± 2.52 ^c^	290 ± 13.75 ^a^
* Methionine	379 ± 25.11 ^b^	462 ± 26.23 ^c^	316 ± 27.68 ^a^
Total non-essential amino acids	11,242	15,935	9461
Total essential amino acids	7679	11,645	6492
Total amino acids	18,921	27,581	15,953

Note: Values followed by different letters denote significant difference at *p* < 0.05 for each analyte of spinach, kale, and purslane across rows; those followed by the same letters denote no significant difference. * Indicates essential amino acid.

**Table 4:** Vitamin content in spinach, kale, and purslane [10] (Mean ± SD; n = 3), dry weight.

Vitamins	Spinach	Kale	Purslane
Vitamin A (Retinol) (IU/100 g)	<100 ± 0 ^a^	<100 ± 0 ^a^	<100 ± 0 ^a^
Vitamin C (Ascorbic acid) (mg/100 g)	51.9 ± 1.5 ^a^	135.0 ± 2.6 ^b^	152 ± 9.3 ^c^
Vitamin E (α-tocopherol) (µg/100 g)	13.5 ± 0.7 ^a^	14.2 ± 0.2 ^a^	11967 ± 57.7 ^b^

Note: Values followed by different letters denote significant difference at *p* < 0.05 for each analyte between the spinach, kale, and purslane across rows; those followed by the same letters denote no significant difference.

**Table 5:** Organic acid content in spinach, kale, and purslane [10] (Mean ± SD; n = 3), dry weight.

Organic Acids	Spinach	Kale	Purslane [10]
Malic acid (mg/100 g)	ND	6138 ± 19.25 ^a^	1155.38 ± 10.72 ^b^
Citric acid (mg/100 g)	1031.94 ± 29.42 ^a^	4826.44 ± 22.55 ^b^	510.08 ± 2.17 ^c^
Oxalic acid (mg/100 g)	8387.47 ± 14.87 ^a^	ND	6757.63 ± 12.83 ^b^

Note: Values followed by different letters denote significant difference at *p* < 0.05 for each analyte between the spinach, kale, and purslane across rows; those followed by the same letters denote no significant difference.

## Data Availability

The data presented in this study are openly available.

## Supplementary Materials

The following are available online, Table S1: Comparison of phytochemicals identified in kale and spinach with previously published purslane phytochemicals.

## Sample Availability

Samples of the compounds are available from the authors.
