# Supplementary material for: Extraction and Natural Bioactive Molecules Characterization in Spinach, Kale and Purslane: A Comparative Study
Source: Molecules. 2021 Apr 26;26(9):2515. doi: 10.3390/molecules26092515 (PMC8123472; doi:10.3390/molecules26092515)
Supplement: Supplementary file 1 [file molecules-26-02515-s001.zip › molecules-1189163-supplementary.pdf]

Supporting Information

Comparative analysis of phytochemical and nutritional contents of leafy greens

Boris Nemzer[a,b], Fadwa Al-Taher[a]*, and Nebiyu Abshiru[a]

[a]*VDF FutureCeuticals, Inc, Momence, IL 60954, USA*
[b]*University of Illinois at Urbana-Champaign, Urbana, IL 61801, USA*

*Corresponding author: Fadwa.al-taher@futureceuticals.com

Supplementary Table S1: Comparison of compounds identified in kale, spinach, and purslane

Identified compounds in eachs sample are marked by "+"
RT - Retention time in minutes
I, II, III... X - isoform number
m/z MS - precursor mass-to-charge ratio
m/z MS/MS -fragment mass-to-charge ratio
ND - not detected
***Data obtained from Nemzer et al. (2020)

Id#	Tentative compound names	m/z MS	on typ	RT (min	Spinach	Kale	Purslane***	m/z MS/MS
**Phenolic & Organic acids**								
1	Dihydroxybenzoic Acid-I	153.0183	M-H	10.0	ND	ND	+	65, 87, 109, 123
2	Dihydroxybenzoic Acid-II			10.4	+	+	+	
3	Dihydroxybenzoic Acid-III			17.5	ND	+	+	
4	Dihydroxybenzoic acid-hexose-I	315.0714		6.4	+	+	+	109, 153
5	Dihydroxybenzoic acid-hexos-II			8.9	+	+	+	
6	Dihydroxybenzoic acid-hexos-III			12.0	+	+	ND	
7	Dihydroxybenzoic acid -hexose dimer	631.1507		9.0	+	+	ND	109, 153, 315
8	Dihydroxybenzoic acid-pentoside-I	285.0609		15.8	ND	ND	+	108, 153
9	Dihydroxybenzoic acid-pentoside-II			15.2	+	ND	+	
10	Dihydroxybenzaldehyde-I	137.0231		15.2	+	+	+	93, 109, 119
11	Dihydroxybenzaldehyde-II			28.2	+	+	+	
12	Dihydroxybenzaldehyde-III			6.6	+	+	ND	
13	Malic acid	133.0130		1.9	+	+	+	71, 89, 115
14	Oxaloacetic acid	130.9971		2.4	ND	ND	+	68.9
15	Tartaric acid	149.0086		1.7	ND	ND	+	
16	Rosmaric acid	359.0770		28.6	ND	ND	+	72, 135, 161, 179, 197, 253
17	Gallic acid-I	169.0133		5.4	+	+	+	70, 123, 125, 151
18	Gallic acid-hexose-I	331.0665		6.7	ND	ND	+	125, 149, 168, 169, 313, 331
19	Gallic acid-hexose-II			9.1	ND	ND	+	
20	Vanillic acid	167.0336		8.4	+	+	+	123
21	Citric acid	191.0186		2.7	+	+	+	85,87, 102, 111, 129, 173
22	Glucaric acid	209.0291		1.6	+	ND	+	85, 103, 133, 147, 191
23	Quinic acid	191.0551		1.7	+	+	+	59, 85, 147, 155, 161 173
24	Gluconic acid	195.0504		1.7	+	+	+	75, 99, 129, 159, 177
25	Sinapoyl glucoside-I	385.1135		22.0	ND	+	ND	164, 205, 223
26	Sinapoyl glucoside-II			22.6	ND	+	ND	
27	Syringic acid-I	197.0444		5.9	ND	ND	+	123, 135, 142, 153, 179, 197
28	Syringic acid-II			9.0	ND	+	+	
29	Syringic acid-III			6.9	ND	ND	+	
30	Syringic acid-IV			9.0	ND	ND	+	
31	Syringic acid-hexose-I	359.0979		9.1	ND	ND	+	123, 135, 179, 197, 359
32	Syringic acid-hexose-II			12.7	+	+	+	
33	Syringic acid-hexose-III			16.8	+	ND	+	
34	Syringic acid-hexose-IV			16.9	ND	ND	+	
35	1-Hydroxybenzoate-O-glucoside	299.0776		6.6	+	+	ND	93, 137, 164,
36	2-Hydroxybenzoate-O-glucoside			11.2	+	+	ND	
37	3-Hydroxybenzoate-O-glucoside			13.6	+	+	ND	
38	4-Hydroxybenzoate-O-glucoside			18.1	+	+	ND	
39	Isopropylmalic acid-I	175.0598		8.5	ND	ND	+	85, 113, 115, 157
40	Isopropylmalic acid-II			10.8	+	ND	+	
41	Isopropylmalic acid-II			13.0	+	ND	+	
42	Isopropylmalic acid-IV			15.0	+	+	+	

Supplementary Table 1 (continued)

Phenolic & Organic acids (continued)

43	Isopropylmalic acid-V		M-H	16.7	+	+	+	
44	Caffeic acid	179.0342		21.1	+	+	+	135
45	Dihydrocaffeic acid-hexose-I	343.1026		14.2	ND	ND	+	138, 163, 181
46	Dihydrocaffeic acid-hexose-II			14.5	ND	ND	+	
47	Caffeic acid-hexose-I	341.0869		15.2	ND	ND	+	135, 179
48	Caffeic acid-hexose-II			16.7	ND	+	+	
49	Caffeic acid-hexose-III			20.3	+	+	+	
50	Caffeic acid-hexose-IV			21.9	+	+	+	
51	Caffeic acid-hexose-V			15.1	ND	ND	+	
52	Caffeic acid-hexose-VI			19.5	ND	ND	+	
53	Caffeoylmalic acid-I	295.0452		23.8	+	ND	+	119, 133, 179
54	Caffeoylmalic acid-II			23.9	+	ND	+	
55	Caffeoylisocitric acid -I	353.0510		10.8	ND	ND	+	161, 173, 179, 191, 209
56	Caffeoylisocitric acid -II			11.6	ND	ND	+	
57	Caffeoylisocitric acid -III			13.5	ND	ND	+	
58	Caffeoylisocitric acid -IV			15.2	ND	ND	+	
59	Caffeoylisocitric acid -V			17.8	ND	ND	+	
60	Caffeoylisocitric acid -VI			18.7	ND	ND	+	
61	Caffeoylisocitric acid -VII			21.7	ND	ND	+	
62	Caffeoylisocitric acid -VIII			22.2	ND	ND	+	
63	Caffeoylisocitric acid -IX			23.8	ND	ND	+	
64	Caffeoylisocitric acid -X			25.2	ND	ND	+	
65	Caffeoylglucaric acid -I	371.0614		6.5	ND	ND	+	147, 161, 179, 191, 209
66	Caffeoylglucaric acid -II			8.3	ND	ND	+	
67	Caffeoylglucaric acid -III			9.5	ND	ND	+	
68	Caffeoylglucaric acid -IV			10.8	ND	ND	+	
69	Caffeoylglucaric acid -V			11.6	ND	ND	+	
70	Caffeoylglucaric acid -VI			12.5	ND	ND	+	
71	Caffeoylglucaric acid -VII			15.3	ND	ND	+	
72	Caffeoylglucaric acid -VIII			16.4	ND	ND	+	
73	Caffeoyltartaric acid-I	311.0403		18.9	ND	ND	+	119, 135, 149, 179, 220
74	Caffeoyltartaric acid-II			20.1	ND	ND	+	
75	Ferulic acid	193.0492		25.7	+	+	+	87, 111, 134, 149, 178
76	Isoferrulic acid			26.6	+	+	+	
77	Ferulic acid-hexose-I	355.1028		19.3	+	+	+	134, 149, 178, 193, 209, 240
78	Ferulic acid-hexose-II			21.6	+	+	+	
79	Ferulic acid-hexose-III			21.7	+	+	+	
80	Ferulic acid-hexose-IV			22.2	ND	ND	+	
81	Ferulic acid-hexose-V			25.7	ND	ND	+	
82	1,2-Diferuloylgentionbiose	693.2053		28.9	ND	+	ND	175, 193
83	Feruloylisocitric acid -I	367.0664		21.2	ND	ND	+	175, 191, 193, 233, 261
84	Feruloylisocitric acid -II			21.8	ND	ND	+	
85	Feruloylisocitric acid -III			22.7	ND	ND	+	
86	Feruloylisocitric acid -IV			23.1	ND	ND	+	
87	Feruloylisocitric acid -V			24.9	ND	ND	+	
88	Feruloylisocitric acid -VI			25.5	ND	ND	+	
89	Feruloylisocitric acid -VII			25.7	ND	ND	+	
90	Feruloylisocitric acid -VIII			27.0	ND	ND	+	
91	Feruloylisocitric acid -IX			27.9	ND	ND	+	
92	Feruloylmalic acid-I	309.0608		26.7	+	+	+	115, 133, 149, 178, 193
93	Feruloylmalic acid-I			26.9	+	+	+	
94	Feruloyltartaric acid-I	325.0560		21.0	ND	ND	+	87, 103, 134, 149, 193
95	Feruloyltartaric acid-II			21.3	ND	ND	+	
96	Feruloyltartaric acid-III			24.6	+	ND	+	
97	Feruloyltartaric acid-IV			23.7	ND	ND	+	
98	Feruloylglucaric acid -I	385.0769		13.6	ND	ND	+	147, 173, 191, 193, 209
99	Feruloylglucaric acid -II			16.5	ND	ND	+	
100	Feruloylglucaric acid -III			18.5	ND	ND	+	

Supplementary Table 1 (continued)

**Phenolic & Organic acids (continued)**								
101	Feruloylglucaric acid -IV		M-H	19.6	ND	ND	+	
102	Feruloylglucaric acid -V			19.7	ND	ND	+	
103	Feruloylglucaric acid -VI			20.4	ND	ND	+	
104	Feruloylglucaric acid -VII			21.4	ND	ND	+	
105	Feruloylglucaric acid -VIII			22.0	ND	ND	+	
106	Coumaric acid	163.0389		20.2	+	+	+	91, 119
107	Coumaric acid-hexose	325.0924		20.2	+	+	+	119, 163, 289
108	Coumaroylmalic acid-I	279.0505		25.6	+	ND	+	119, 133, 163
109	Coumaroylmalic acid-II			25.9	+	ND	+	
110	Coumaroyltartaric acid	295.0454		23.6	+	ND	+	119, 149, 163, 217
111	Coumaroylisocitric acid -I	337.0562		20.1	ND	ND	+	119, 129, 147, 163, 173
112	Coumaroylisocitric acid -II			21.4	ND	ND	+	
113	Coumaroylisocitric acid -III			21.5	ND	ND	+	
114	Coumaroylisocitric acid -IV			22.5	ND	ND	+	
115	Coumaroylisocitric acid -V			24.2	ND	ND	+	
116	Coumaroylisocitric acid -VI			24.5	ND	ND	+	
117	Coumaroylisocitric acid -VII			24.9	ND	ND	+	
118	Coumaroylisocitric acid -VIII			25.6	ND	ND	+	
119	Coumaroylisocitric acid -IX			26.2	ND	ND	+	
120	Coumaroylisocitric acid -X			27.3	ND	ND	+	
121	Coumaroylglucaric acid -I	355.0663		9.8	ND	ND	+	163, 209, 191, 133
122	Coumaroylglucaric acid -II			12.6	ND	ND	+	
123	Coumaroylglucaric acid -III			14.2	ND	ND	+	
124	Coumaroylglucaric acid -IV			16.6	ND	ND	+	
125	Coumaroylglucaric acid -V			16.8	ND	ND	+	
126	Coumaroylglucaric acid -VI			18.3	ND	ND	+	
127	Coumaroylglucaric acid -VII			19.9	ND	ND	+	
128	Coumaroylglucaric acid -VIII			20.8	ND	ND	+	
**Chlorogenic acid**								
129	Caffeoylquinic acid derivative	353.0875	M-H	12.8	ND	+	+	135, 179, 191
130	3-Caffeoylquinic acid			14.2	ND	+	ND	135, 179, 191
131	5-Caffeoylquinic acid			19.2	ND	+	+	135, 173, 179, 191
132	4-Caffeoylquinic acid			22.4	ND	+	ND	191
133	5-Feruloylquinic acid			20.5	ND	+	ND	134, 191, 193
134	4-Feruloylquinic acid			20.7	ND	+	ND	134, 193
135	3-coumaroylquinic acid	337.0934		18.5	ND	+	ND	119, 163, 191
136	5-coumaroylquinic acid			22.0	ND	+	ND	163, 173, 191
137	4-coumaroylquinic acid			24.3	ND	+	ND	191
**Flavonoid**								
138	Naringenin I	271.0604	M-H	34.1	ND	+	+	119, 151, 227, 271
139	Naringenin II	271.0604		33.6	ND	ND	+	
140	Rutin	609.1455		24.6	ND	ND	+	300, 301
141	Quercetin	301.0348		31.9	ND	+	+	151, 179, 273, 301
142	Quercetin galactoside	463.0874		25.7	ND	+	+	179, 255, 271, 300, 301
143	Quercetin galactoside			25.9	ND	+	+	
144	Quercetin glucoside-I			27.8	ND	ND	+	151, 179, 273, 301
145	Quercetin glucoside-II			28.7	ND	ND	+	
146	Quercetin-diglucoside-I	625.1400		23.8	ND	+	+	151, 178, 301, 463
147	Quercetin-diglucoside-II			24.3	ND	+	+	
148	Quercetin malonyl-galactoside	549.0873		27.1	ND	ND	+	255 300, 301, 463, 505
149	Quercetin malonyl-glucoside			27.6	ND	ND	+	179, 273, 301, 463, 505

Supplementary Table 1 (continued)

Flavonoid (continued)

150	Quercetin acetyl-galactoside	505.0980		27.1	ND	ND	+	255, 271, 300, 301, 463
151	Quercetin acteyl-glucoside			27.7	ND	ND	+	179, 273, 301, 463, 505
152	Kaempferol-I	285.0397		31.7	ND	ND	+	107, 151, 183, 255, 285
153	Kaempferol-II			35.2	ND	+	+	
154	Kaempferol glucoside-I			26.8	ND	+	+	
155	Kaempferol glucoside-II	447.0915		27.5	ND	+	+	227, 255, 285 (284), 447
156	Kaempferol glucoside-III			27.2	ND	ND	+	
157	Kaempferol pentose-I	417.0826		29.5	ND	ND	+	255, 284, 285, 417
158	Kaempferol pentose-II			28.6	ND	ND	+	
159	Catechin-I	289.0704		20.2	ND	ND	+	151, 179, 205, 245
160	Catechin-II			22.7	ND	ND	+	
161	Isorhamnetin-I	315.0503		29.7	ND	ND	+	164, 197, 211, 300, 315
162	Isorhamnetin-II			35.5	ND	+	+	
163	Isorhamnetin glucoside-I	477.1030		27.7	+	+	+	151, 299, 314, 315, 477
164	Isorhamnetin glucoside-II			27.9	ND	+	+	
165	Isorhamnetin diglucoside	639.1563	M-H	24.3	+	+	+	285, 313, 315, 476, 477, 639
166	Quercetin hexose-derivative-I	871.3810		26.8	ND	ND	+	161, 301, 391, 463
167	Quercetin hexose-derivative-II			28.8	ND	ND	+	
168	Isorhamnetin-3-O-gentiobioside	639.1581		27.3	+	+	ND	315, 477
169	Isorhamnetin-3-O-sophoroside-7-O-D-glucosid	639.1575		25.0	ND	+	ND	
170	Isorhamnetin 3-*O*-sophoroside-7-*O*-glucoside	801.2095		22.4	ND	+	ND	315, 477, 639
171	Kaempferol 3-O-Sophoroside	609.1461		24.9	ND	+	ND	283, 285, 447
172	Kaempferol 3-O-gentiobioside	609.1461		27.0	ND	+	ND	283, 285, 447
173	Quercetin 3-*O*-(feruloyl)sophoroside-7-*O*-glucoside-I	963.2412		20.9	ND	+	ND	191, 284, 446, 609, 801
174	Quercetin 3-*O*-(feruloyl)sophoroside-7-*O*-glucoside-II			21.6	ND	+	ND	
175	Quercetin-3-O-sophoroside-7-O-D-glucoside	787.1953		19.4	ND	+	ND	
176	Kaempferol 3-*O*-sophoroside-7-*O*-glucoside	771.1989		20.0	ND	+	ND	283, 284, 446, 609
177	Kaempferol 3-*O*-(sinapoyl) sophoroside-7-*O*-glucoside	977.2568		22.0	ND	+	ND	283, 284, 285, 446,
178	Kaempferol 3-*O*-(caffeoyl)sophoroside-7-*O*-glucoside	933.2306		21.3	ND	+	ND	284, 285, 446, 609, 771
179	Kaempferol 3-*O*-(*p*-coumaroyl)sophoroside-7-*O*-glucoside	917.2357		22.6	ND	+	ND	
180	Kaempferol 3-*O*-(feruloyl)sophoroside-7-*O*-glucoside	947.2463		22.4	ND	+	ND	485, 446, 591, 609, 785

Alkaloids

181	Oleraceins A-I			21.8	ND	ND	+	
182	Oleraceins A-II	504.1492		23.7	ND	ND	+	85, 119, 147, 196, 229
183	Oleraceins A-III			25.3	ND	ND	+	
184	Oleraceins B-I			22.4	ND	ND	+	
185	Oleraceins B-II	534.1600		24.1	ND	ND	+	177, 196, 243, 287, 372
186	Oleraceins B-III			25.7	ND	ND	+	
187	Oleraceins C-I			20.8	ND	ND	+	
188	Oleraceins C-II	666.2013	M+H	21.8	ND	ND	+	85, 119, 147, 196, 229
189	Oleraceins C-III			23.7	ND	ND	+	
190	Oleraceins D-I	696.2119		21.4	ND	ND	+	
191	Oleraceins D-II			22.4	ND	ND	+	196, 243, 287, 372
192	Oleraceins D-III			24.1	ND	ND	+	
193	Oleracein N/S-I	842.2487		26.0	ND	ND	+	127, 147, 177, 339
194	Oleracein N/S-II			26.4	ND	ND	+	
195	Oleracein O-I	872.2595		25.7	ND	ND	+	127, 147, 177, 207, 339

Supplementary Table 1 (continued)

**Alkaloids (continued)**								
196	Oleracein O-II			26.3	ND	ND	+	
197	Oleracein O-III			26.6	ND	ND	+	
198	Oleracein p	828.2542		20.8	ND	ND	+	127,147,196,309, 504
199	Oleracein Q	858.2649		21.4	ND	ND	+	85,145,177
200	Oleracein U-I		M+H	23.7	ND	ND	+	70, 86,119,147,177,343
201	Oleracein U-II	342.0966		25.3	ND	ND	+	
202	Oleracein U-III			27.2	ND	ND	+	119,147,163,182,300
203	Oleracein W-I			22.6	ND	ND	+	86,163,177,359
204	Oleracein W-II	358.0916		23.9	ND	ND	+	
205	Oleracein W-III			25.5	ND	ND	+	127,153,163,177,299
**Betacynins**								
206	Betanin	551.1497	M+H	11.7	ND	ND	+	389
**Glucosinolates**								
207	Glucoraphanin-I	436.0406		2.4	ND	+	ND	96, 111, 178, 372
208	Glucoraphanin-II			2.8	ND	+	ND	
209	Sinigrin	358.0266		3.3	ND	+	ND	96, 161, 195
210	Progoitrin-I	388.0372		2.4	ND	+	ND	96, 111, 195, 216
211	Progoitrin-II			2.9	ND	+	ND	
212	4-Hydroxyglucobrassicin	463.0481	M-H	10.2	ND	+	ND	96, 169, 221, 267
213	Glucobrassicin	447.0532		20.4	ND	+	ND	128, 167, 206, 208
214	Methoxyglucobrassicin-I	477.0638		23.4	ND	+	ND	96, 161, 195, 259
215	Methoxyglucobrassicin-II			26.4	ND	+	ND	
216	Gluconasturtiin	422.0556		2.2	ND	+	ND	96, 195, 259, 277, 337
217	Neoglucobrassicin	477.0646		23.0	ND	+	ND	96, 259, 274
**Amino acid**								
218	Aspartic acid	132.0291		1.6	+	+	ND	71, 88, 114, 115
219	Asparagine	131.0451		1.6	+	+	ND	71, 88, 95, 114, 115
220	Glutamine	145.0607	M-H	1.6	+	+	ND	74, 84, 109, 127
221	Glutamic acid	146.0445		1.7	+	+	ND	84, 102, 109, 127, 128
222	Histidine	154.0611		1.6	+	+	ND	–
223	Phenylalanine	166.0861		6.7	+	+	+	120, 166
224	Tryptophan	205.0974	M+H	14.3	+	+	ND	146, 188
225	Tryptophan-NH3	188.0708		14.3	+	+	ND	146
**Vitamin**								
226	Pantothenic acid	218.1034		8.8	+	+	ND	88, 146
227	Niacin/nicotinic acid	124.0393	M-H	2.0	+	+	ND	80, 96, 106
228	Riboflavin/Vitamon B2	377.1451		22.7	+	+	ND	243
229	Ascorbic acid	175.0237		2.1	+	+	ND	87, 115
**Other compounds**								
230	Dopamine	154.0858		2.7	+	ND	+	91, 107, 119, 137
231	Trigonellin	138.0548		1.7	+	+	+	94, 138
232	Adenosine	268.1034		4.0	+	+	+	136, 250, 268
233	Noradrenaline	170.0807		1.8	ND	ND	+	107, 152, 171
234	5'-Methylthioadenosine	298.0963		15.2	+	+	ND	136
235	Glutathione-I			3.2	+	ND	ND	162, 177, 179, 231, 291
236	Glutathione-II	307.0909	M+H	3.5	+	ND	ND	
237	Glutathione dervative-III			2.5	+	ND	ND	
238	Glutathione-dimer I	613.1592		3.3	+	+	ND	231, 265, 288, 308, 355
239	Glutathione-dimer II			3.5	+	+	ND	
240	Feruloyl-gentiobiose	501.1603		28.6	+	+	+	177.05
241	Feruloyltyramine	314.1380		29.3	ND	ND	+	121, 145, 163
242	Isoferuloyltyramine			30.5	ND	ND	+	
